# NAFLD/MAFLD: New Evidence

**DOI:** 10.3390/ijms24087241

**Published:** 2023-04-14

**Authors:** Alessandro Mantovani, Andrea Dalbeni

**Affiliations:** 1 Section of Endocrinology, Diabetes and Metabolism, Department of Medicine, University and Azienda Ospedaliera Universitaria Integrata of Verona, 37126 Verona, Italy; 2 Section of General Medicine C and Liver Unit, University and Azienda Ospedaliera Universitaria Integrata of Verona, 37126 Verona, Italy

The aim of the second edition of our Special Issue, entitled *“Nonalcoholic Fatty Liver Disease/Metabolic Associated Fatty Liver Disease: New Insights 2.0”*, is to report the recent experimental and observational studies about NAFLD/MAFLD, which is the most common chronic liver disease worldwide to date [1]. Overall, nine original articles and eight narrative reviews have been published. Among original articles, Mantovani et al. showed that the presence of Fibroscan^®^-assessed significant fibrosis was independently associated with an increased risk of developing worse glycaemic control in female patients with type 2 diabetes (T2DM) and NAFLD/MAFLD [2]. These findings further support the assertion that the relationship between NAFLD/MAFLD and T2DM is complex and bidirectional [3]. In addition, the study by Mantovani et al. also highlights the need for a multidisciplinary and holistic approach to patients with T2DM and NAFLD [4]. Viganò et al. reported that the FIB-4 score might be suboptimal to identify patients with advanced forms of NAFLD/MAFLD referring to liver centers, given that the Authors found that approximately one-fifth of patients included in the study were false negative at FIB-4 score, despite a liver stiffness measurement ≥ 8kPa [5]. Therefore, maybe, the use of more techniques to identify (non-invasively) patients with NAFLD/MAFLD and liver fibrosis might be more appropriate in clinical practice. In another observational study, Bourgonje et al. documented that higher plasma calprotectin levels were independently associated with suspected NAFLD/MAFLD, as well as with an increased risk of all-cause mortality [6]. Although further studies are needed, these results might suggest that circulating levels of calprotectin might be a potential and novel biomarker of NAFLD/MAFLD. The other original articles published in our Special Issue discussed other important issues about NAFLD/MAFLD, such as the adipokines and cytokines in the saliva of obese people with NAFLD/MAFLD [7], the relationship between FXR (farnesoid X receptor) transcriptional activity and the mTORC1 (mammalian target of rapamycin1)/S6K1 (ribosomal protein S6 kinase beta-1)/PGC1α (peroxisome proliferator receptor gamma coactivator1α) pathway [8], the association between liver alterations and insulin resistance in pediatric patients with NAFLD/MAFLD [9], the role of ERMCs (endoplasmatic reticulum–mitochondria contacts) in the progression of advanced forms of NAFLD/MAFLD [10], the role of mitochondrial electron transport chain (ETC) [11] and histone lysine demethylase KDM7A (also called JHDM1D) [12] in the development of NAFLD/MAFLD.

Among narrative reviews, Teng et al. discussed the recent information about the pathological progression of NAFLD/MAFLD and its advanced forms, as well as the potential therapeutic targets [13], including GLP-1 receptor agonists and other glucose-lowering agents. In this regard, Subramanian et al. discussed the cellular crosstalk between hepatic stellate cells and other cells within the liver, able to promote liver fibrosis in NAFLD/MAFLD [14]; biological mechanisms that should be used for the development of additional drugs in order to treat NAFLD/MAFLD. The other narrative reviews published in our Special Issue discussed other relevant issues about NAFLD/MAFLD, including the role of fibroblast growth factors [15], hydrogen sulfide [16], nuclear receptors [17], calcium signaling [18] in the development and progression of NAFLD/MAFLD, the role of various natural products in the treatment of NAFLD/MAFLD [19] as well as the association between MAFLD and chronic kidney disease (CKD) [20].

We hope that the second edition of the Special Issue will help produce new evidence about NAFLD/MAFLD, in order to improve the lives of these people.
